# Assessment of African swine fever vaccine candidate ASFV-G-∆MGF in a reversion to virulence study

**DOI:** 10.1038/s41541-023-00669-z

**Published:** 2023-05-29

**Authors:** Paul Deutschmann, Jan-Hendrik Forth, Julia Sehl-Ewert, Tessa Carrau, Elisenda Viaplana, Jose Carlos Mancera, Alicia Urniza, Martin Beer, Sandra Blome

**Affiliations:** 1 Friedrich-Loeffler-Institut, Suedufer 10, 17493 Greifswald, Insel Riems Germany; 2 Zoetis Manufacturing and Research Spain, Finca la Riba, Carretera de Camprodon s/n, 17813 L’Hostalnou de Bianya, Girona, Spain; 3 Zoetis Belgium, Mercuriusstraat 20, 1930 Zaventem, Belgium

**Keywords:** Live attenuated vaccines, Virology

## Abstract

African swine fever (ASF) has gained panzootic dimensions and commercial vaccines are still unavailable. Recently, a series of live attenuated vaccines has raised hope for an efficacious and safe vaccine, among them “ASFV-G-∆MGF”. We tested the latter in an in vivo reversion to virulence study in accordance with *International Cooperation on Harmonisation of Technical Requirements for Registration of Veterinary Medicinal Products* guidelines. Upon forced animal passaging, a virus variant emerged that was associated with transient fever and an increased replication and shedding. However, all animals were healthy upon completion of the study and reversion to significant virulence was not observed. The genomic changes did not affect the recombination site but involved deletions and reorganizations in the terminal regions of the genome. Thus, our study underscores that in-depth safety characterization is needed for live ASF vaccines. For this particular candidate, additional studies should target long-term effects and transmission characteristics before thorough benefit-risk analysis can be carried out.

## Introduction

African swine fever (ASF) has recently spread in panzootic dimensions, exerting an immense pressure on the global pig industry and at the same time endangering entire populations of rare wild pig species^1^. The disease is caused by ASF virus (ASFV), a member of the genus *Asfivirus* within the family *Asfarviridae*^2^. Outside its sylvatic cycle in sub-Saharan Africa, the disease is characterized by a haemorrhagic fever with high lethality in domestic and wild suids, which represent the only susceptible mammals^3^. Following an introduction into Georgia in 2007, ASF spread successively through eastern and central Europe, most of Asia, and recently to the Caribbean^4–6^. Without vaccines, the available control measures have failed to eliminate the disease in most countries affected by ASF^4^. Thus, the call for a safe and efficacious vaccine is louder than ever, and research efforts to find solutions have recently intensified. Still, only a few attempts have produced successful vaccine candidates that have gone beyond proof-of-concept studies. To date, live attenuated vaccines (LAV) are the most promising concept, since complete protection against lethal field strains have only been shown with this group of vaccines^7^. While reports in peer-reviewed publications of the first efficacious LAV prototypes raise hope for a licensable product on the horizon, there are still significant concerns with their safety^8^. In particular, the inevitable ability of live vaccines viruses to replicate may result in genetic mutations and adaption in target tissues.

In the present study, we evaluated vaccine candidate “ASFV-G-∆MGF”^9,10^, from here on called “ΔMGF”), a genetically modified LAV that has shown a most promising efficacy profile, in a standard in vivo reversion to virulence study in naïve weaner pigs. In short, the vaccine virus was passaged five times in domestic pigs in accordance with *VICH (International Cooperation on Harmonisation of Technical Requirements for Registration of Veterinary Medicinal Products) guideline 41 for the examination of live veterinary vaccines in target animals for absence of reversion to virulence* (reference number EMA/CVMP/VICH/1052/2004).

## Results

### Clinical Observations

**Fig. 1:**
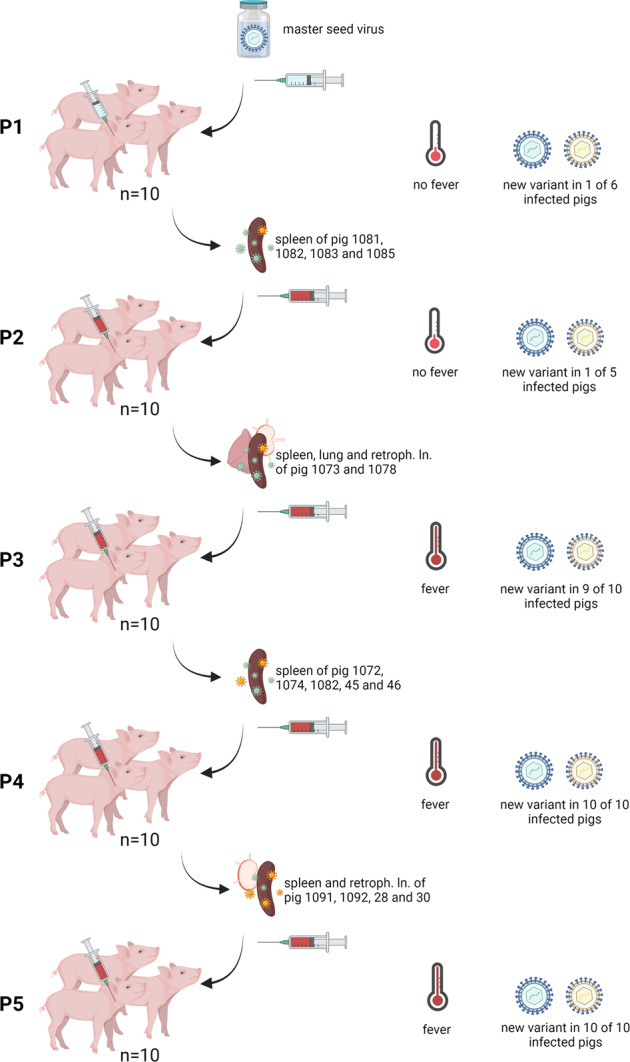
Study design. Shown are the number of animals per passage (P1 to P5) on the left side, and the tissues used for further passaging. Passage materials are depicted as organs in the center. Clinical and virological results throughout the study are visualized on the right side as fever occurrence and variant detection. Created with BioRender.com.

No clinical abnormalities or fever were observed during the first animal passage (see Fig. 1). In the second animal passage, however, three pigs displayed a transient rise in body temperatures up to 40.4 °C. Beginning from passage three and in both subsequent passages, high fever to a maximum of 42.1 °C (pig #31, P4, 6 dpv, supplementary Figure 1) was observed in numerous animals with a peak at around five and six dpi. In passage five, a body temperature of 41 °C or above was recorded in nine out of ten animals for at least one day. Elevated body temperatures were clinically mirrored by mild to moderate signs of lacking appetite and apathy, which were scored to a maximum of three cumulative clinical score points in a single animal in passage four (animal #31, 6 dpi). All other clinical observations between passages three and five were very mild and resulted in only one cumulative clinical score point. In passage five, with an extended observation period of 21 dpi, normalization of body temperatures and the subsequent disappearance of clinical abnormalities were observed after the fever peak. Animals were clinically healthy at the end of the 21-day observation period of passage five, with the exception of animal #1110, which showed transient lameness (not related to ASFV) on 14 and 15 dpi, recovering thereafter. One animal (#21) displayed a complete loss of sensory and motoric function of the front right leg immediately after inoculation. Treatment of the clinically diagnosed peripheral nerve injury with dexamethasone did not result in any improvement of the lameness, so the animal was euthanized for ethical reasons on 7 dpi.

### Pathological findings

All pigs were subjected to detailed pathological examination. Overall, very few lesions were detectable in all passages, i.e. slightly enlarged lymph nodes and pulmonary consolidation. No correlation or significant difference was observed when comparing lesions with passage level or time point after inoculation, i.e. day 7 versus day 21 (data not further shown).

### Detection of ASFV genome

**Fig. 2:**
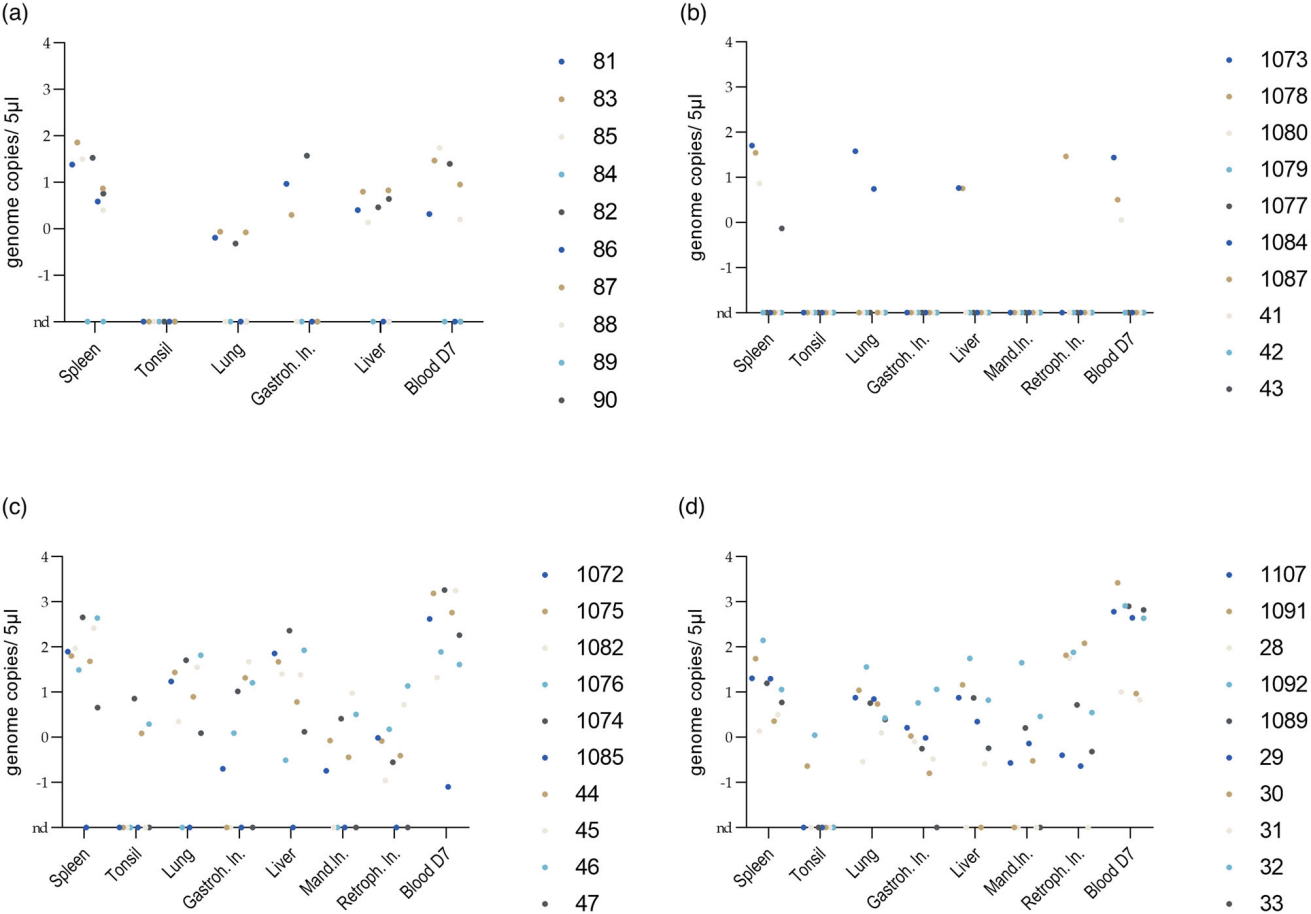
Genome loads detected in different tissues during passages 1-4. The figure shows the genome loads (log10) in the different organ samples and in EDTA blood at day 7 after inoculation of different passages (passage 1 (**a**), passage 2 (**b**), passage 3 (**c**) and passage 4 (**d**)). Individual animals are represented by colored dots (see legend).

**Fig. 3:**
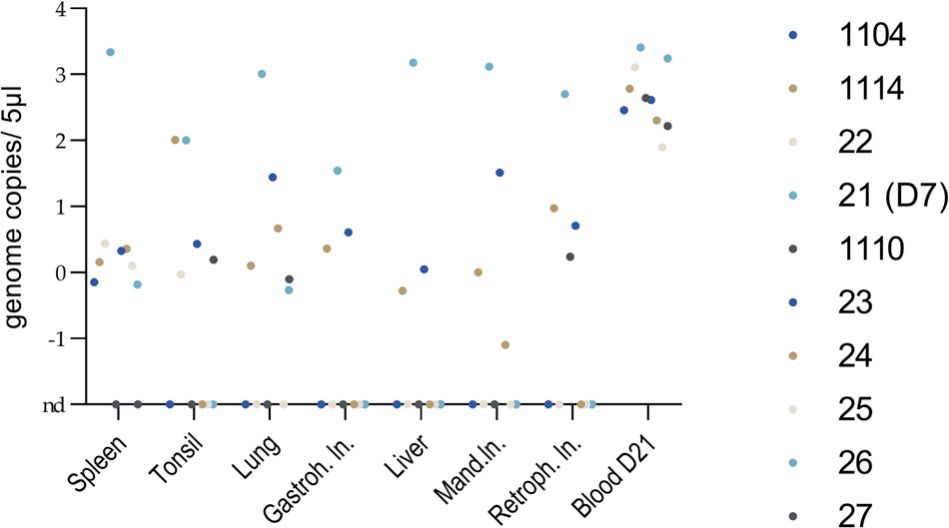
Genome loads detected in different tissues in the final passage 5. The figure shows the genome loads (log10) in different organ samples and in EDTA blood at day 21 (day 7 for pig #21) after inoculation with passage 5. Individual animals are represented by colored dots (see legend).

qPCR screening of the samples taken from the first passage yielded less than 7.2 × 10¹ ASFV gc per 5 μl template from the entire sample set (shown in Fig. 2). In two pigs, the vaccine virus could not be detected. In passage two, no more than 5 × 10¹ gc were detected in a single sample and in five pigs, the vaccine virus was not detected at all. In the third passage, however, all pigs were positive in at least one sample and up to 1.8 × 10^3^ gc were quantified. All pigs were positive for ASFV genome on passage four with a maximum 2.6 × 10^3^ gc in a sample. In passage five, again, all animals were positive for ASFV genome, however with slightly lower genome loads in the different tissues but comparable loads in blood to the previous passages (prolonged monitoring phase of 21 dpi, see Fig. 3). In pig #21, up to 2.6 × 10^3^ gc were detected (euthanized on 7 dpi).

Investigation of different swab samples showed low viral genome loads in seven out of ten animals in at least one type of swab on seven dpi (a maximum of 4 gc in a sample). At 14 dpi, six out of nine animals were positive and three out of nine were positive 21 dpi. Interestingly, higher genome loads were detected from swabs at 21 dpi than in the weeks before (see Supplementary Table 1).

### Whole-genome sequencing

**Fig. 4:**
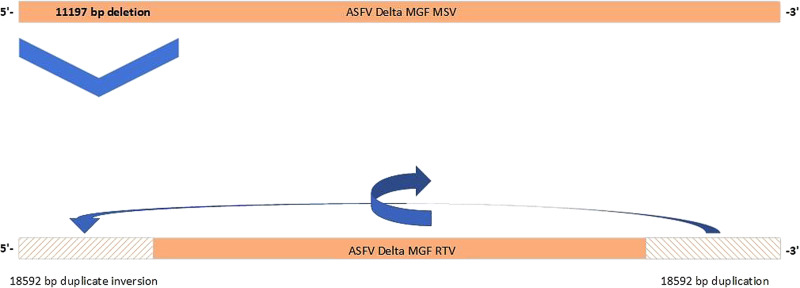
Genetic changes in the variant virus. The genome of “ΔMGF” is depicted in the upper part, and the genome of the “ΔMGFnv” at the bottom. Changes include a 11197 bp deletion at the 5’-end of the genome (left hand side) and a duplication of 18592 bp from the 3’-end of the genome which are bound to the 5’-end in reverse complementary orientation (arrows at the lower part).

In two samples of passage 4, an ASFV variant (named “ΔMGFnV”) was detected characterized by a large deletion at the 5’-end of the genome. This deletion of 11,197 bp leads to the loss of 18 previously annotated ASFV genes (see Fig. 4 and Table 1 for detailed and functional findings). Interestingly, the deletion is accompanied by a duplication of 18,592 bp from the 3’-end of the genome which are bound to the 5’-end in reverse complementary orientation leading to the duplication of 29 genes. With respect to the genetically modified regions in multigene families 360 and 505, i.e. genes MGF505-1R, MGF360-12L, -13L, and -14L, MGF505-2R, and MGF505-3R, the vaccine candidate has remained stable over all passages. The same applies for the core genome of the vaccine candidate.

**Table 1:** List of modified genes found in the ΔMGFnV genome.

Gene	Type	Minimum	Maximum	Function
MGF 360-1La CDS	Deletion	163	2463	Unknown
MGF 360-1Lb CDS	Deletion	2391	2711	Unknown
MGF 360-2 L CDS	Deletion	2797	3885	Unknown
KP177R CDS	Deletion	4026	4559	P22, structural protein, transmembrane domain
L83L CDS	Deletion	4696	4941	Early gene, located in cytoplasm, interacts with IL1B, non-essential
L60L CDS	Deletion	5042	5,2	Unknown
MGF 360-3 L CDS	Deletion	5359	6429	Unknown
MGF 110-1 L CDS	Deletion	6822	7466	Early gene. Membrane protein. Might play a role in virus cell tropism, and may be required for efficient virus replication in macrophages (inferred from homology).
ASFV G ACD 00090 CDS	Deletion	7465	7578	Unknown
MGF 110-2 L CDS	Deletion	7646	796	Early gene. Might play a role in virus cell tropism, and may be required for efficient virus replication in macrophages (inferred from homology).
MGF 110-3 L CDS	Deletion	8057	8431	Unknown
ASFV G ACD 00120 CDS	Deletion	8542	8766	Unknown
MGF 110-4 L CDS	Deletion	8745	9119	Early gene, causes the redistribution of luminal ER protein to an enlarged ERGIC compartment. Glycosylated.
MGF 110-5L-6L CDS	Deletion	9308	9925	Early gene, might play a role in virus cell tropism, and may be required for efficient virus replication in macrophages (inferred from homology).
MGF 110-7 L CDS	Deletion	10,132	10,545	Glycosylated, might plays a role in virus cell tropism, and may be required for efficient virus replication in macrophages (inferred from homology).
285 L CDS	Deletion	10,86	11,144	Transmembrane domain.
DP60R CDS	Deletion	232	396	Transmembrane domain, glycosylated.
ASFV G ACD 01990 CDS	Deletion	517	663	Unknown
ASFV G ACD 01980 CDS	Duplication	1354	1548	Unknown
MGF 360-21 R CDS	Duplication	1537	2607	Might play a role in virus cell tropism, and may be required for efficient virus replication in macrophages (inferred from homology).
ASFV G ACD 01960 CDS	Duplication	3053	3184	Unknown
MGF 360-19Rb CDS	Duplication	3253	3522	Might play a role in virus cell tropism, and may be required for efficient virus replication in macrophages (inferred from homology).
MGF 360-19Ra CDS	Duplication	3536	4345	
ASFV G ACD 01940 CDS	Duplication	4498	4656	Unknown
DP96R CDS	Duplication	4956	5246	Unknown
DP71L CDS	Duplication	5345	5557	Interacts with the host phosphatase PP1 catalytic subunit (PPP1CB) and recruits it to dephosphorylate EIF2S1/eIF2alpha and therefore restores the host translation that has been shut-down by the host. Also inhibits the EIF2S1/eIF2alpha-ATF4-DDIT3/CHOP pathway.
MGF 360-18 R CDS	Duplication	5,54	6253	Might play a role in virus cell tropism, and may be required for efficient virus replication in macrophages (inferred from homology).
L11L CDS	Duplication	6483	6764	Membrane protein.
I10L CDS	Duplication	7003	7515	Structural protein, in virion, Transmembrane protein. In viral envelope.
I9R CDS	Duplication	7591	7881	Transmembrane protein
ASFV G ACD 01870 CDS	Duplication	7844	7981	Unknown
I8L CDS	Duplication	8076	8387	Nonessential. Unknown.
hypthetical CDS	Duplication	8434	8541	Unknown
I7L CDS	Duplication	8601	8909	Transmembrane protein
MGF 100-3 L CDS	Duplication	9008	9316	Might play a role in virus cell tropism, and may be required for efficient virus replication in macrophages (inferred from homology).
MGF 100-1 L CDS	Duplication	9681	10,106	Unknown
MGF 505-11 L CDS	Duplication	10,225	11,853	Might play a role in virus cell tropism, and may be required for efficient virus replication in macrophages (inferred from homology).
MGF 360-16 R CDS	Duplication	11,933	12,991	Might play a role in virus cell tropism, and may be required for efficient virus replication in macrophages (inferred from homology).
DP238L CDS	Duplication	13,168	13,884	Unknown
I196L CDS	Duplication	13,978	14,586	Late gene. Unknown.
I177L CDS	Duplication	14,579	15,112	Late gene. Single pass membrane protein. Glycosylated.
I215L CDS	Duplication	15,153	15,791	Early and late gene. Accepts ubiquitin from the E1 complex and catalyzes its covalent attachment to other proteins. Performs the second step in the ubiquitination reaction that targets specifically a protein for degradation via the proteasome. By controlling the ubiquitination status of specific host proteins, the virus may target them to degradation and thereby optimize the viral replication. Knockdown impairs viral infection, with lower number of synthesized viral genomes and lower viral progeny.
I329L CDS	Duplication	16,08	17,069	Late gene. Single-pass type I membrane protein. Highly glycosylated.
I73R CDS	Duplication	17,279	17,497	Unknown
I243L CDS	Duplication	17,581	18,312	Late gene. Transcription factor S-II-related protein
DP60R CDS	Duplication	416	580	Transmembrane domain, glycosylated
ASFV G ACD 01990 CDS	Duplication	701	847	Unknown

#### Screening for the novel virus variant ΔMGFnV

Using tailored qPCR, the emergence of the novel virus variant ΔMGFnV was tracked back to pig #1081 of passage one, the spleen of which was part of the organ pool for subsequent passaging (see supplementary Table 3). In this animal, a mixed infection of the MSV and ΔMGFnV was observed, while in six other pigs only the wild-type MSV was detected. In the second passage, the ΔMGFnV was detected in two pigs (#1080 monoinfection, #1073 mixed infection with MSV), while three pigs were positive for just the MSV. In passage three, all positive pigs harbored the ΔMGFnV, one individual (#1082) as a monoinfection and nine other animals as mixed infection with the MSV. The picture remained similar after the fourth passage: All pigs were positive for ΔMGFnV, one of which by monoinfection and nine out of ten were also infected with the MSV. In passage five, ΔMGFnV was detected as a monoinfection in four pigs, while the other six animals were coinfected with both the MSV and the variant.

### Comparative growth kinetics

The purity of the respective ΔMGF and ΔMGFnV isolates as well as the presence of both isolates in the coinfected cell culture was confirmed by tailored qPCR. After incubation of the virus suspensions (0 hpi), titers between 10^2.75^ (MSV + nV, nv) and 10³ HAD_50_/ml (MSV) were detected from supernatants in HAT (see supplementary Table 4). The increase of titers developed uniformly with a maximum logarithmic deviation of 0.5 at a single timepoint. Viruses grew up to 10^6.75^ HAD_50_/ml (MSV and nV monoinfecton) and 10^7^ HAD_50_/ml (coinfection).

When testing samples from the kinetics in the tailored qPCR, for the FAM channel (detection of ΔMGFnV), a mean deviation of 2.98 % was recorded from ASFV genome detection in virotype 2.0. For the HEX channel (nV detection), the mean deviation from virotype 2.0 was 8.55% (shown in supplementary Table 4). Variant ΔMGFnV reached cq values of roughly 17 in mono- and coinfection at 48 and 72 hpi, while the growth of the nV variant yielded cq values of roughly 19 (monoinfection) or 21 (coinfection), at the respective times. Considering the beforementioned deviations in the tailored qPCR, the growth kinetics of both isolates developed quite uniformly.

## Discussion

As the global spread of ASF continues, the situation for pig holders and nature conservationists has never been as tense as it is now. Millions of pigs are at risk, representing the livelihoods of farmers, and entire species of certain wild suids, e.g. bearded pigs, that are now threatened with extinction^11^. Against this background, but without neglecting the need for additional measures and integrated control schemes^4^, it is widely accepted that a vaccine is urgently needed to complement available control measures^12^. In this context, we may not be in the position to wait for a perfect vaccine candidate and should rather stress the application of a practicable solution as fast as reasonably possible. On the other hand, we cannot afford to compromise on vaccine safety, or as Gavier-Widen, et al.^13^ put it, allow hasty solutions. Experiences in Spain and Portugal from the last century using attenuated field isolates^14^, and possibly very recently in China (https://www.reuters.com/article/us-china-swinefever-vaccines-insight-idUSKBN29R00X visited May 15^th^ 2022), show us that premature field testing of live vaccines can cause prolonged forms of ASF with extended shedding and delayed clinical characteristics, often below the detection limit. The use of vaccine viruses with unacceptable residual virulence or that revert to virulence can lead to an iatrogenic, self-sustaining infection cycle with increasing virulence. This would further complicate eradication efforts and these scenarios must be avoided under all circumstances.

Consequently, we took one of the few fully efficacious and possibly licensable vaccine candidates, “ΔMGF”, and examined its safety profile in terms of genetic stability and reversion to virulence under a worst-case scenario.

While both the core genome and the genetically modified regions of the vaccine candidate remained stable over the entire study, we observed the occurrence of a virus variant (“ΔMGFnV”) in one animal of passage one that subsequently overgrew the wild-type MSV. This virus variant harbors significant changes in the variable regions at the 5’ and 3’ end of the viral genome. Over the course of the experiment, coinfections with the wild-type MSV and the “ΔMGFnV” occurred at an increasing rate, and in passage five, “ΔMGFnV” was detected as a monoinfection in four pigs (see Supplementary Table 3). No clinical difference was observed between animals coinfected with both variants (MSV or ΔMGFnV) or infected with just one variant (see Supplementary Fig. 1). Clinically, the variant was associated with a slightly increased virulence, e.g. induction of a short episode of raised body temperature or fever in most animals in the later passages. However, all animals, even in the final 5^th^ passage, were clinically inapparent at the end of the experiment, showed no evidence of incipient, chronic infection, and showed little or no vaccine virus in the tissues tested. When evaluating the transient occurrence of fever during the later passages, it must be considered that with more effective replication, inoculum doses for the subsequent passages increased. However, for the ΔMGF MSV, infection doses similar to what was back titrated from the inocula used in this study (maximum of 10^5.75^ HAD_50_/ml in passage 4) induced no clinical signs clearly attributable to the inoculation.

To further explore whether the observed genomic changes in “ΔMGFnV” were indeed responsible for the increased replication competency in vivo, we conducted comparative in vitro growth kinetics of both viruses in primary macrophages, revealing no indications for a significant advantage in in vitro replication of the variant virus in this setup. Clear limits in explanatory power should be considered; however, since modifications in the MGF regions are known to have effects on interferon expression^15^, the full consequences of such changes may only be observable in vivo. When comparing interferon induction in PBMC-derived macrophages inoculated with ΔMGF MSV or ΔMGFnV (by real-time RT-PCR), respectively, no reactions were seen that would suggest a stronger suppression of interferon induction by the variant virus (data not further shown). The underlying factors causing the in vivo replication advantages of ΔMGFnV remain therefore unanswered, stressing that many of these questions can only be addressed by further safety and efficacy studies in the target species due to the highly complex virus-host interactions of ASFV. Those studies will have to include immune responses against the new variant.

Whether our findings showing genetic changes outside the regions that were modified to achieve viral attenuation and a slight rise in virulence after in vivo passaging disqualify the vaccine candidate is a matter of critical debate, since the mode of transmission is highly artificial (selecting particularly positive samples for further passaging and intramuscular injection of tissue homogenates), and there is no evidence of reversion to the original levels of high virulence of ASFV “Georgia07”. In this context, it could be discussed whether a brief period of fever can be tolerable for an efficacious and therefore otherwise practicable first-generation ASFV vaccine. A prerequisite for this assumption would be that the novel variant is genetically stable and does not mark the beginning of a maintained process of further genetic adaption, only mirrored by this first mutation.

In this context, the mutant detection qPCR described here was also used retrospectively for representative samples from several efficacy tests with the ΔMGF vaccine candidate, with clearly negative results for the variant “ΔMGFnV” (data not further shown).

It should be noted, however, that in this case a viral variant with altered geno- and phenotypic properties has already emerged in the first passage, i.e. after the application of the MSV. This phenomenon did not occur in any of the previous studies, but it is relevant because it could also happen in the field during intensive use. It is also remarkable that the mechanism of a large deletion in the terminal regions of the ASFV genome complemented by reorganization of genomic regions has been observed for an ASFV strain under natural circumstances before^16^. The respective virus, ASFV “Estonia 2014”, showed an attenuated phenotype and has been used for several animal trials thereafter e.g.^17,18^. No further genetic changes were observed upon passaging in domestic and wild suids. We may have unraveled a common mechanism of ASFV for genetic adaption when a certain selection pressure is applied.

Any future use of the vaccine candidate has to be based on a thorough benefit-risk assessment considering all safety and efficacy features as well as the potential vaccination scenario. Along these lines, further studies with the evolved virus variant are needed, which should address excretion, long-term effects, and transmission to naive contact animals.

In general, our study confirms that even the most promising ASF live vaccine candidates require very comprehensive safety testing (Gavier-Widen et al., 2020). However, it also provides a first indication of what an attenuated ASF vaccine virus would need to do to increase its replication efficacy in the animal or to compensate for deletions in the MGF region. This knowledge can be deepened and used to devise strategies to make these changes even more difficult for the virus.

If field application is considered after benefit-risk-assessment, one should apply genetic tools to differentiate infected from vaccinated animals (genetic DIVA). The PCR described here could aid such approaches. Moreover, conditional licensing under controlled conditions could be a solution to obtain field data for final decisions on the use of the vaccine to complement national control measures.

## Methods

### Experimental design

The VICH guideline 41 (https://www.ema.europa.eu/en/vich-gl41-target-animal-safety-examination-live-veterinary-vaccines-target-animals-absence-reversion, visited April 27th 2023) states that the study is to be carried out using the master seed at the maximum release titer expected in the recommended dose for five serial passages in target animals. The time interval between inoculation of the animals and harvest for each passage must be justified based upon the characteristics of the test organism. Moreover, the most sensitive class, age, sex and serological status of animals should be used. At least two animals are to be used for the first four groups and a minimum of eight for the fifth group. The initial administration and subsequent passages shall be carried out using a recommended route of administration or natural route of infection that is the most likely to lead to reversion to or increase in virulence and result in recovery of the organism following replication in the animal. Passage inocula should be collected and prepared from the most likely source of the spread of the organism.

The above-mentioned recommendations were implemented as follows: Dose and route of inoculation were chosen to maximize the chance of reversion to or increase in virulence, representing a worst-case scenario of vaccine virus transmission. Considering this, undiluted master seed virus (MSV) was used and day 7 was set as timepoint for organ and blood collection for passaging. At this point, recovery of vaccine virus had the highest chance based on our experience from previous studies^10^. Along the same lines, an intramuscular transfer of material was chosen to maximize the chance of infection given the experience that the parenteral route is much more efficient than an oral or oro-nasal inoculation (Guinat, Gogin et al. 2016). Given the low detection rate in previous trials, the study was performed in groups of ten, 6-10-week-old weaned naïve pigs which were obtained from the breeding unit of the Friedrich-Loeffler-Institute (FLI) in Mariensee, Germany, and moved to the high containment facilities of the FLI on the Isle of Riems, Germany. Before inoculation, the blood of each pig was collected in an EDTA tube for reference purposes. During the experiment, the rectal body temperature was recorded and health status based on a clinical score was assessed daily. At the respective day of study completion, a full pathological examination was performed based on the modified protocol published by Galindo-Cardiel^19^ as previously described^17^. Blood as well as tissue samples, including spleen, lung, liver, tonsil, kidney, salivary gland and gastro-hepatic, and mandibular lymph nodes were collected upon necropsy. Pigs of the first four passages were observed for seven days postinoculation (dpi). Passage five was observed for 21 dpi, and oral, nasal and rectal swabs were collected weekly to trace vaccine virus shedding during the last passage. The animal experiment was approved by the State Office for Agriculture, Food Safety and Fishery in Mecklenburg-Western Pomerania (LALFF M-V) under reference number 7221.3-1-020/21.

### Cells and titrations

Titrations and virus isolations were performed on porcine peripheral blood mononuclear cell (PBMC) derived macrophages obtained from the EDTA-treated blood of a healthy donor pig. In brief, Pancoll animal, Density: 1.077 g/ml (PAN-biotech) was used for separation of the erythrocytes by mixture at 1:10 with the blood. After resting for 2 h, the clear supernatant was diluted 1:1 with PBS and cleared by centrifugation at 800 x g. The supernatant was discarded and the cell pellet resuspended with PBS; these washing steps were repeated three times. Finally, the cell pellet was resuspended in cell culture medium and counted using a TC20 automated cell counter (Bio-Rad) with trypan blue staining. For titrations, cells were seeded in 96-well culture plates (Primaria; Corning) with 100 μl/well at a density of 5 × 10^6^ cells/ml and cultivated for 48 h using GM-CSF as a growth factor (2 ng/ml). 100 μl of the respective samples diluted in cell culture medium at factors 10^−1^ to 10^−8^ was added to each well for endpoint titration. 20 μl of a 1 % suspension of erythrocytes from the same donor pig in PBS was added after 24 h. For determination of titers, infected wells were read 48 and 72 h-post-infection using the hemadsorption as readout. The HAD_50_ was calculated according to the method by REED and MUENCH^20^.

### Passaging of the virus

A pure pre-master master seed virus grown on primary swine macrophages was prepared by USDA-ARS at the Plum Island Animal Disease Center and transferred to Zoetis. Master seed virus grown on a proprietary cell line used in collaboration with Aptimmune Biologics Inc known to support the growth of ASFV was subsequently prepared by Zoetis and provided to the FLI ready to use. The transfer of the vaccine virus from macrophages to the permanent cell culture did not require visible adaptations steps as there was no apparent change in the virus phenotypically, in growth kinetics and virus yields. Moreover, the cytopathic effect was similar. The new mutations that appeared in pigs were absent in the MSV material used for pig inoculation, as demonstrated by PCR (Elisenda Viaplana, Zoetis, personal communication). Compared with the virus grown on macrophages, the virus propagated on permanent cells has two point mutations in the B438L gene. One of these mutations is silent (position 98378, A → G), the other leads to an amino acid exchange (position 98378, C → G; alanine → proline). In a previously published animal trial, no differences were observed in safety and efficacy^10^.

Five groups of ten pigs each were inoculated intramuscularly with 1 ml of the respective virus suspension into the right side of the neck using 2-ml syringes with 21 G cannulas.

Group one received the “ΔMGF” MSV at a dose of 1.75 ×10^6^ HAD_50_/ml. Organs were sampled after each passage and screened by qPCR for the presence of ASFV genome. For subsequent passages, tissues with the highest genome loads were selected, pooled at equal proportions, and homogenized in PBS at 20 Hz for 30 seconds in a grinding jar set compatible with the TissueLyser II (QIAGEN) to receive a 10 % tissue suspension. A total of 1.5 g of tissue was weighed and suspended in 13.5 ml of PBS for the preparation of each inoculum. After centrifugation at 1800 x g for 5 minutes, supernatants were obtained and administered to the following passage group. Tissue homogenate from passage one was back titrated to 10^4.25^ HAD_50_/ml. The subsequent inocula contained 10^2.25^ (P2), 10^4^ (P3) and 10^5.75^ HAD_50_/ml (P4). In detail, the inoculum for P2 contained spleen materials of animals #1081, #1082, #1083, and #1085. For P3, the spleen and lung of animal #1073 and the spleen and retropharyngeal lymph node of animal #1078 were pooled. Spleen materials of animals #1072, #1074, #1082, #45, an #46 were pooled. For the final passage, spleen materials originated from animals #1091 and #1092, pooled with retropharyngeal lymph nodes of animals #28 and #30. The choice always reflected materials with the lowest cq values. An overview of the study design and the organs chosen for passaging is provided in Fig. 1.

### Preparation of samples and ASFV qPCR

Tissue samples were homogenized in 1 ml phosphate buffered saline (PBS) with a metal bead on a TissueLyser II (QIAGEN) at 30 Hz for 3 min, then centrifuged at 14,000 rpm for 5 minutes. Swab samples were soaked in the medium for 1 h, then thoroughly vortexed and aliquoted. All samples were stored at −80 °C or immediately processed.

Nucleic acids were extracted using the NucleoMag Vet Kit (Machery-Nagel) on the KingFisher® extraction platform (Thermo Scientific). qPCR for the detection of ASFV genome was either conducted according to the protocol published by King, et al.^21^ or with commercial virotype 2.0 ASFV (Indical Bioscience) on C1000™ thermal cyclers with the CFX96™ Real-Time System (Bio-Rad). An in-house ASFV full genome standard was employed for the calculation of genome copies (gc) and harmonization between runs, using six ASFV full genome dilution steps containing between 1 × 10^0^ and 1 × 10^5^ gc, which were tested along with each qPCR run and set as genome standard in the Bio-Rad software.

### Whole-genome sequencing

For whole-genome sequencing, a minimum of 100 ng of DNA was sent to and sequenced by Eurofins Genomics. This service included the preparation of a 450 bp DNA sequencing library using a modified version of the NEBNext Ultra™ II FS DNA Library Prep Kit for Illumina and sequencing on an Illumina NovaSeq 6000 with S4 flowcell, XP workflow and in PE150 mode (Illumina).

### Data analysis

The sequence data received from Eurofins Genomics was quality trimmed and mapped against a previously produced MSV whole-genome sequence as reference using Newbler 3.0 (Roche) with default parameters. Mapped reads were extracted and assembled using SPAdes 3.13 in the mode of error correction prior to assembly and standard parameters. The resulting contigs were mapped against the ASFV MSV reference sequence and the contigs were curated and assembled manually in Geneious Prime. For validation of the assembly and mean coverage determination, all reads were mapped against the final contig again using Newbler 3.0 with default parameters.

### Tailored qPCR

For the identification of the novel ASFV variant, two qPCRs were designed using Geneious Prime (Biomatters) spanning the reorganization site at the 5’ end (probes directly positioned at the reorganization site) with one specifically recognizing the ΔMGF MSV (FAM-labeled) and one recognizing the ΔMGF novel variant, from here called ΔMGFnV (HEX-labeled) (see Table 1 and Supplementary Table 3).

### Growth kinetics

The novel virus variant ΔMGFnV was isolated from the blood of pig #22 of the fifth passage and cultivated on macrophages in T25 cell culture flasks (Primaria; Corning) and titrated as described in the 2.2 Cells and titrations section. The absence of the MSV virus was assured by tailored qPCR. Growth kinetics were also conducted on macrophages obtained as described above. Cells were counted using a Bio-Rad TC 20 Automated Cell Counter with trypan blue staining, counting only living cells. T25 flasks were infected at a multiplicity of infection of 0.1 with either the ΔMGF MSV, the ΔMGFnV or both viruses simultaneously. After 2 h of incubation, medium supernatants were removed, cells were rinsed once with PBS- and flasks were resuspended with cell culture medium. 300 μl of supernatant were removed at -2 h postinfection (hpi, before adding the virus solution), 0 hpi (after incubation), and 4, 8, 12, 24, 48 and 72 hpi and immediately stored at −80 °C. Samples were analyzed by the commercial ASFV real-time PCR system virotype 2.0 (Indical Bioscience GmbH) and tailored qPCR for differentiation between ΔMGF MSV and ΔMGFnV replication, and by titration on macrophages using the methods described above.

### Reporting summary

Further information on research design is available in the Nature Research Reporting Summary linked to this article.

## Supplementary information

Supplemantary Files

REPORTING SUMMARY

## Data Availability

All relevant data to support the findings described in the text are included in the main text or in the supplementary materials. Additional data is available from the corresponding author upon reasonable request.
