# Supplementary material for: Assessment of African swine fever vaccine candidate ASFV-G-∆MGF in a reversion to virulence study
Source: NPJ Vaccines. 2023 May 29;8:78. doi: 10.1038/s41541-023-00669-z (PMC10227017; doi:10.1038/s41541-023-00669-z)
Supplement: Supplementary file 1 — Supplemantary Files [file 41541_2023_669_MOESM1_ESM.pdf]

Supplementary figure 1: daily body temperature of individual animals during the respective passages

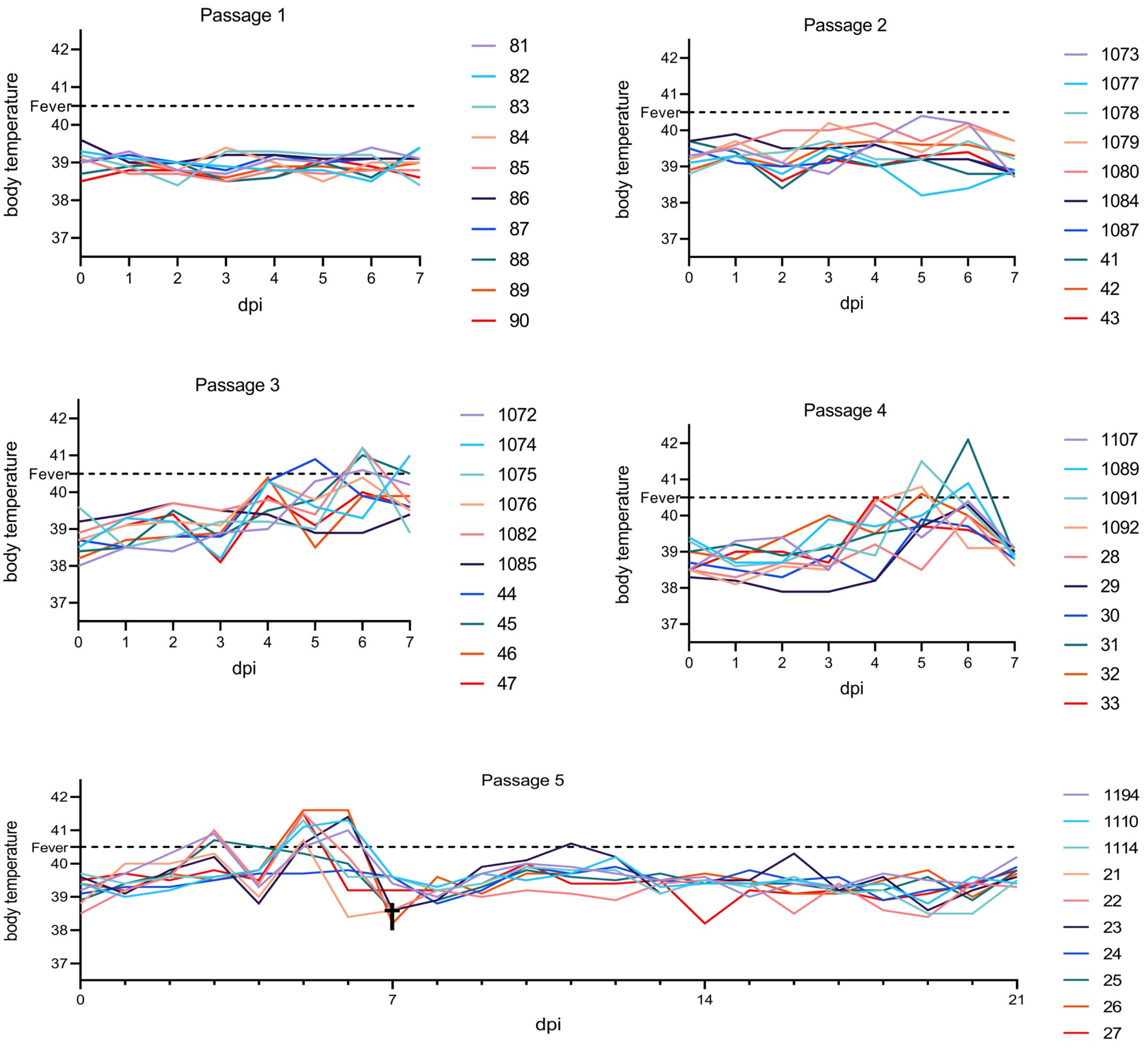

Supplementary Table 1: Detection of viral genome from swabs

King PCR (Detection of ASFV genome copies / 5µl)

ASF Syst1 FAM, **D7 pv.**

	1104	1110	1114	21*	22	23	24	25	26	27
nasal swab	1.326E+00	N/A	N/A	2.424E+00	4.429E+00	N/A	N/A	N/A	4.548E-01	N/A
pharyng. Swab	2.327E+00	2.598E+00	N/A	N/A	N/A	N/A	2.357E+00	N/A	4.020E+00	3.735E-01
rectal swab	N/A	N/A	N/A	2.587E+00	N/A	N/A	N/A	N/A	N/A	N/A

ASF Syst1 FAM, **D14 pv.**

	1104	1110	1114	21 †	22	23	24	25	26	27
nasal swab	N/A	N/A	N/A		N/A	N/A	N/A	N/A	N/A	1.441E-01
pharyng. Swab	N/A	1.174E+00	8.686E-01		N/A	1.722E+00	5.726E-02	N/A	2.307E+00	N/A
rectal swab	N/A	N/A	N/A		N/A	N/A	N/A	N/A	N/A	N/A

ASF Syst1 FAM, **D21 pv.**

	1104	1110	1114	21 †	22	23	24	25	26	27
nasal swab	N/A	N/A	N/A		N/A	N/A	N/A	N/A	N/A	N/A
pharyng. Swab	N/A	N/A	1.830E+01		N/A	3.151E+00	4.003E+01	N/A	N/A	N/A
rectal swab	N/A	N/A	N/A		N/A	N/A	N/A	N/A	N/A	N/A

Supplementary Table 2: Primers and Probes for tailored qPCR

Oligo type	Target	Name	Sequence	Length
Primer	MSV	ASFV_MSV_for2	CTCAAGAAGCATGATGTAGCCTT	206 bp
Primer	MSV	ASFV_MSV_rev2	TCCAACATCCCATACTTATCCACA	
Probe	MSV	ASFV_MSV_probe	5’-FAM- AGGCCCTCACTATCTTTAATGAAGGAG-3’-BHQ1	
Primer	MGFnV	ASFV_RTV_for1	AGCGCACGGCAAACATCAAC	213 bp
Primer	MGFnV	ASFV_RTV_rev1	TCCAACATCCCATACTTATCCACA	
Probe	MGFnV	ASFV_RTV_Probe	5’-HEX-CAAGGTTTCGGAGGAATGAAGGAGT-3’-BHQ1	

Supplementary Table 3: Results from tailored qPCR for variant detection

Passage 1

King PCR (Detection of ASFV Genome)

	81	82	83	84	85	86	87	88	89	90
spleen	30,45	29,89	28,6	n.d.	30	33,53	32,45	34,24	n.d.	32,86
tonsil	n.d.	n.d.	n.d.	n.d.	n.d.	n.d.	n.d.	n.d.	n.d.	n.d.
lung	36,56	37,02	36,04	n.d.	n.d.	n.d.	36,08	n.d.	n.d.	n.d.
gastrohep. ln	32,04	29,7	34,64	n.d.	n.d.	n.d.	n.d.	n.d.	n.d.	n.d.
liver	34,24	34	32,71	n.d.	35,26	n.d.	32,59	n.d.	n.d.	33,31
blood d7pv	34,57	30,38	30,12	n.d.	29,05	n.d.	32,1	35,01	n.d.	n.d.

Tailored PCR (Differentiation between MSV and novel "RtV"-variant)

FAM (MSV variant)

	81	82	83	84	85	86	87	88	89	90
spleen	33,17	30,3	29,92	n.d.	37,16	n.d.	33,29	35,42	n.d.	33,35
tonsil	37,37	n.d.	n.d.	n.d.	n.d.	n.d.	n.d.	n.d.	n.d.	n.d.
lung	n.d.	34,91	34,34	n.d.	n.d.	n.d.	34,72	n.d.	n.d.	37,19
gastrohep. ln	33,19	31,16	36,26	n.d.	n.d.	n.d.	n.d.	n.d.	n.d.	n.d.
liver	n.d.	33,36	35,27	n.d.	n.d.	n.d.	38,31	37,04	n.d.	36,13
blood d7pv	37,07	31,75	32,75	n.d.	n.d.	n.d.	34,56	34,4	n.d.	n.d.

HEX (RtV variant)

	81	82	83	84	85	86	87	88	89	90
spleen	31,08	n.d.	n.d.	n.d.	n.d.	n.d.	n.d.	n.d.	n.d.	n.d.
tonsil	n.d.	n.d.	n.d.	n.d.	n.d.	n.d.	n.d.	n.d.	n.d.	n.d.
lung	n.d.	n.d.	n.d.	n.d.	n.d.	n.d.	n.d.	n.d.	n.d.	n.d.
gastrohep. ln	36,84	n.d.	n.d.	n.d.	n.d.	n.d.	n.d.	n.d.	n.d.	n.d.
liver	35,46	n.d.	n.d.	n.d.	n.d.	n.d.	n.d.	n.d.	n.d.	n.d.
blood d7pv	37,07	n.d.	n.d.	n.d.	n.d.	n.d.	n.d.	n.d.	n.d.	n.d.

Passage 2

King PCR (Detection of ASFV Genome)

	1073	1077	1078	1079	1080	1084	1087	41	42	43
spleen	31,75	n.d.	32,38	n.d.	35,08	n.d.	n.d.	n.d.	n.d.	39,03
tonsil	n.d.	n.d.	n.d.	n.d.	n.d.	n.d.	n.d.	n.d.	n.d.	n.d.
lung	32,25	n.d.	n.d.	n.d.	n.d.	35,56	n.d.	n.d.	n.d.	n.d.
gastrohep. ln	n.d.	n.d.	n.d.	n.d.	n.d.	n.d.	n.d.	n.d.	n.d.	n.d.
liver	35,49	n.d.	35,52	n.d.	n.d.	n.d.	n.d.	n.d.	n.d.	n.d.
Mand. Ln.	n.d.	n.d.	n.d.	n.d.	n.d.	n.d.	n.d.	n.d.	n.d.	n.d.
Retroph. Ln.	n.d.	n.d.	32,69	n.d.	n.d.	n.d.	n.d.	n.d.	n.d.	n.d.
blood D7	33,44	n.d.	33,89	n.d.	35,47	n.d.	n.d.	n.d.	n.d.	n.d.

Tailored PCR (Differentiation between MSV and novel "RtV"-variant)

FAM (MSV variant)

	1073	1077	1078	1079	1080	1084	1087	41	42	43
spleen	33,38	n.d.	32,21	n.d.	n.d.	n.d.	n.d.	n.d.	n.d.	37,21
tonsil	n.d.	n.d.	36,75	n.d.	n.d.	n.d.	n.d.	n.d.	n.d.	n.d.
lung	n.d.	n.d.	38,39	n.d.	n.d.	33,49	n.d.	n.d.	n.d.	n.d.
gastrohep. ln	n.d.	n.d.	36,61	n.d.	n.d.	n.d.	n.d.	n.d.	n.d.	n.d.
liver	37,64	n.d.	34,56	n.d.	n.d.	35,46	n.d.	n.d.	n.d.	n.d.
Mand. Ln.	n.d.	n.d.	n.d.	n.d.	n.d.	n.d.	n.d.	n.d.	n.d.	n.d.
Retroph. Ln.	n.d.	n.d.	32,17	n.d.	n.d.	n.d.	n.d.	n.d.	n.d.	n.d.
blood D7	n.d.	n.d.	34,65	n.d.	n.d.	n.d.	n.d.	n.d.	n.d.	n.d.

HEX (RtV variant)

	1073	1077	1078	1079	1080	1084	1087	41	42	43
spleen	32,05	n.d.	n.d.	n.d.	33,88	n.d.	n.d.	n.d.	n.d.	n.d.
tonsil	n.d.	n.d.	n.d.	n.d.	n.d.	n.d.	n.d.	n.d.	n.d.	n.d.
lung	33,3	n.d.	n.d.	n.d.	n.d.	n.d.	n.d.	n.d.	n.d.	n.d.
gastrohep. ln	37,05	n.d.	n.d.	n.d.	n.d.	n.d.	n.d.	n.d.	n.d.	n.d.
liver	35,29	n.d.	n.d.	n.d.	n.d.	n.d.	n.d.	n.d.	n.d.	n.d.
Mand. Ln.	n.d.	n.d.	n.d.	n.d.	n.d.	n.d.	n.d.	n.d.	n.d.	n.d.
Retroph. Ln.	n.d.	n.d.	n.d.	n.d.	n.d.	n.d.	n.d.	n.d.	n.d.	n.d.
blood D7	28,09	n.d.	n.d.	n.d.	35,53	n.d.	n.d.	n.d.	n.d.	n.d.

Passage 3

King PCR (Detection of ASFV Genome)

	1072	1074	1075	1076	1082	1085	44	45	46	47
spleen	29,02	26,35	29,36	30,44	28,75	n.d.	29,77	27,19	26,4	33,36
tonsil	n.d.	32,68	n.d.	43,99	n.d.	n.d.	35,37	43,33	34,64	n.d.
lung	31,33	29,69	30,63	43,55	34,46	n.d.	32,53	30,24	29,3	35,35
gastrohep. ln	38,17	32,11	43,78	35,36	n.d.	n.d.	31,06	29,81	31,44	n.d.
liver	29,16	27,38	29,82	37,44	30,76	n.d.	32,94	30,82	28,92	35,24
Mand. Ln.	38,27	34,22	35,93	n.d.	n.d.	n.d.	37,22	32,24	33,91	43,49
Retroph. Ln.	35,72	37,63	35,97	35,06	39,04	n.d.	37,13	33,14	31,7	n.d.
blood D7	26,48	24,24	24,5	29,03	31,04	39,59	25,99	24,29	30,03	27,73

Tailored PCR (Differentiation between MSV and novel "RtV"-variant)

FAM (MSV variant)

	1072	1074	1075	1076	1082	1085	44	45	46	47
spleen	32,05	27,19	n.d.	31,66	n.d.	n.d.	29,18	26,4	26,07	36,16
tonsil	n.d.	32,59	n.d.	36,94	n.d.	n.d.	34,03	n.d.	n.d.	n.d.
lung	34,22	32,42	n.d.	36,77	n.d.	n.d.	32,87	30,47	29,57	n.d.
gastrohep. ln	n.d.	32,1	n.d.	35,23	n.d.	n.d.	31,35	30,03	30,8	n.d.
liver	31,85	30,13	n.d.	n.d.	n.d.	n.d.	32,52	30,9	28,65	n.d.
Mand. Ln.	n.d.	34,51	n.d.	36,58	n.d.	n.d.	36,15	32,21	32,79	n.d.
Retroph. Ln.	n.d.	36,39	36,54	33,61	n.d.	n.d.	36,72	34,34	32,83	n.d.
blood D7	30,69	28,31	n.d.	31,4	n.d.	n.d.	29,13	28,05	31,03	n.d.

HEX (RtV variant)

	1072	1074	1075	1076	1082	1085	44	45	46	47
spleen	29,5	27,17	30,09	31,54	29,28	n.d.	32,83	30,51	31,21	34,02
tonsil	n.d.	n.d.	n.d.	n.d.	n.d.	n.d.	n.d.	n.d.	n.d.	n.d.
lung	31,88	30,17	31,6	n.d.	34,25	n.d.	36,86	33,93	33,27	38,85
gastrohep. ln	n.d.	35,05	n.d.	37,31	38,12	n.d.	35,58	n.d.	n.d.	35,34
liver	29,94	29,01	30,17	n.d.	31,89	n.d.	36,02	34,15	33,56	36,13
Mand. Ln.	n.d.	34,65	33,96	n.d.	n.d.	n.d.	n.d.	36,36	n.d.	37,68
Retroph. Ln.	n.d.	n.d.	37,15	n.d.	n.d.	n.d.	n.d.	36,33	36,34	n.d.
blood D7	26,97	25,28	24,9	30	31,78	n.d.	27,03	26,17	32,9	29,44

Passage 4

King PCR (Detection of ASFV Genome)

	1107	1089	1091	1092	28	29	30	31	32	33
spleen	29,48	29,85	28,02	26,67	33,39	29,52	32,65	32,18	30,31	31,28
tonsil	n.d.	n.d.	36	33,69	n.d.	n.d.	n.d.	n.d.	n.d.	n.d.
lung	30,92	31,32	30,37	28,66	35,63	31,02	31,38	33,53	32,43	32,53
gastrohep. ln	33,13	34,68	33,75	31,3	34,18	33,89	36,53	35,46	30,3	n.d.
liver	30,92	30,93	29,96	28,01	n.d.	32,69	n.d.	35,79	31,1	34,67
Mand. Ln.	35,76	33,16	n.d.	28,33	n.d.	34,3	35,59	n.d.	32,3	n.d.
Retroph. Ln.	35,19	31,45	27,77	27,56	28,02	36	26,9	n.d.	32,03	34,92
blood D7	24,56	24,16	22,43	24,13	30,51	25,01	30,63	31,09	25,05	24,44

Tailored PCR (Differentiation between MSV and novel "RtV"-variant)

FAM (MSV variant)

	1107	1089	1091	1092	28	29	30	31	32	33
spleen	n.d.	34,36	32,11	28,32	n.d.	34,99	n.d.	33,41	32,66	38,02
tonsil	n.d.	n.d.	n.d.	n.d.	n.d.	n.d.	n.d.	n.d.	n.d.	n.d.
lung	n.d.	n.d.	34,18	29,95	n.d.	n.d.	33,65	n.d.	n.d.	n.d.
gastrohep. ln	n.d.	n.d.	n.d.	n.d.	n.d.	n.d.	n.d.	n.d.	34,33	n.d.
liver	n.d.	n.d.	34,8	29,54	35,54	n.d.	n.d.	34,69	33,35	n.d.
Mand. Ln.	n.d.	n.d.	n.d.	30,45	n.d.	n.d.	n.d.	n.d.	36,49	n.d.
Retroph. Ln.	n.d.	32,85	30,52	29,47	29,78	n.d.	29,35	n.d.	34,04	n.d.
blood D7	n.d.	43,75	43,38	26,58	n.d.	40,94	n.d.	32,97	29,02	42,9

HEX (RtV variant)

	1107	1089	1091	1092	28	29	30	31	32	33
spleen	33,17	32,42	31,07	42,66	n.d.	33,16	35,81	n.d.	36,82	36,37
tonsil	n.d.	n.d.	n.d.	n.d.	n.d.	n.d.	n.d.	n.d.	n.d.	n.d.
lung	34,03	34,76	33,98	44,09	n.d.	35,24	n.d.	n.d.	38,39	35,25
gastrohep. ln	37,28	n.d.	n.d.	n.d.	n.d.	n.d.	n.d.	n.d.	n.d.	n.d.
liver	34,24	36	34,55	44,74	n.d.	37,15	37,28	n.d.	37,15	38,81
Mand. Ln.	39,6	36,55	n.d.	n.d.	n.d.	n.d.	n.d.	n.d.	37,95	n.d.
Retroph. Ln.	n.d.	n.d.	n.d.	n.d.	37,02	36,69	n.d.	n.d.	n.d.	n.d.
blood D7	26,17	26,01	24,08	26,37	33,26	26,44	32,04	33,31	26,81	26,13

Passage 5

King PCR (Detection of ASFV Genome)

	1104	1110	1114	21*	22	23	24	25	26	27
spleen	35,85	n.d.	34,75	23,29	33,74	34,14	34,02	34,94	35,97	n.d.
tonsil	n.d.	n.d.	28,09	28,1	35,4	33,76	n.d.	n.d.	n.d.	34,61
lung	n.d.	n.d.	34,94	24,48	n.d.	30,11	32,9	n.d.	36,29	35,68
gastrohep. ln	n.d.	n.d.	34	29,76	n.d.	33,12	n.d.	n.d.	n.d.	n.d.
liver	n.d.	n.d.	36,3	23,86	n.d.	35,15	n.d.	n.d.	n.d.	n.d.
Mand. Ln.	n.d.	n.d.	35,31	24,09	n.d.	29,85	39,17	n.d.	n.d.	n.d.
Retroph. Ln.	n.d.	34,47	31,81	25,57	n.d.	32,77	n.d.	n.d.	n.d.	n.d.
blood D21	26,46	25,79	25,29	23,03	24,12	25,9	27,02	28,47	23,63	27,32
				*D7						

Tailored PCR (Differentiation between MSV and novel "RtV"-variant)

FAM (MSV variant)

	1104	1110	1114	21*	22	23	24	25	26	27
spleen	n.d.	n.d.	n.d.	24,19	n.d.	35,56	37,31	n.d.	n.d.	n.d.
tonsil	n.d.	n.d.	30,82	30,25	n.d.	36,09	n.d.	n.d.	n.d.	n.d.
lung	n.d.	n.d.	36,78	25,1	n.d.	32,64	n.d.	n.d.	n.d.	n.d.
gastrohep. ln	n.d.	n.d.	36,67	31,67	n.d.	36,62	n.d.	n.d.	n.d.	n.d.
liver	n.d.	n.d.	n.d.	25,04	n.d.	n.d.	n.d.	n.d.	n.d.	n.d.
Mand. Ln.	n.d.	n.d.	n.d.	26,22	n.d.	33,26	n.d.	n.d.	n.d.	n.d.
Retroph. Ln.	n.d.	n.d.	n.d.	27,5	n.d.	35,07	n.d.	n.d.	n.d.	n.d.
blood D21	n.d.	n.d.	n.d.	23,72	n.d.	32,83	31,12	35,07	n.d.	32,46
				*D7						

HEX (RtV variant)

	1104	1110	1114	21*	22	23	24	25	26	27
spleen	n.d.	n.d.	n.d.	31,18	n.d.	37,2	n.d.	n.d.	n.d.	n.d.
tonsil	n.d.	n.d.	n.d.	n.d.	n.d.	n.d.	n.d.	n.d.	n.d.	38,26
lung	n.d.	n.d.	n.d.	30,04	n.d.	n.d.	37,16	n.d.	n.d.	n.d.
gastrohep. ln	n.d.	n.d.	n.d.	n.d.	n.d.	n.d.	n.d.	n.d.	n.d.	n.d.
liver	n.d.	n.d.	n.d.	30,8	n.d.	n.d.	n.d.	n.d.	n.d.	n.d.
Mand. Ln.	n.d.	n.d.	n.d.	n.d.	n.d.	n.d.	n.d.	n.d.	n.d.	n.d.
Retroph. Ln.	n.d.	n.d.	35,13	n.d.	n.d.	n.d.	n.d.	n.d.	n.d.	n.d.
blood D21	28,97	28,6	28,19	25,87	26,24	28,8	30,27	32,22	26,12	30,24

Supplementary Table 4: Comparative growth kinetics of MGF MSV and MGF nV

Tailored qPCR for differentiation of MSV and nV, cq values

	MGF MSV				MGF MSV + nV			MGF nV			
	virotype 2.0 FAM	RtV PCR FAM (MSV)	virotype, FAM deviation (%)	RtV PCR HEX (nV)	virotype 2.0 FAM	RtV PCR FAM (MSV)	RtV PCR HEX (nV)	virotype 2.0 FAM	RtV PCR FAM (MSV)	RtV PCR HEX (nV)	virotype, HEX deviation (%)
-2hpi	n.d.	n.d.		n.d.	n.d.	n.d.	n.d.	n.d.	n.d.	n.d.	
0hpi	30,20	31,99	5,93	n.d.	30,05	32,84	33,06	29,68	n.d.	32,33	8,93
4hpi	27,91	28,41	1,79	n.d.	27,79	29,27	30,77	28,18	n.d.	30,67	8,84
8hpi	25,94	26,50	2,16	n.d.	25,21	26,60	28,16	26,32	n.d.	28,39	7,86
12hpi	23,35	24,20	3,64	n.d.	24,22	25,77	27,79	24,68	n.d.	26,73	8,31
24hpi	19,50	20,21	3,64	n.d.	19,92	20,92	23,67	21,51	n.d.	23,31	8,37
48hpi	17,00	17,19	1,12	n.d.	17,42	17,54	20,90	17,89	n.d.	19,39	8,38
72hpi	17,14	17,58	2,57	n.d.	17,25	17,53	20,99	17,54	n.d.	19,15	9,18
		mean deviation:	2,98							mean deviation:	8,55

Genome copies/µl determined by virotype 2.0 ASFV qPCR

	MGF MSV	MGF MSV + nV	MGF nV
-2hpi	n.d.	n.d.	n.d.
0hpi	2,74E+01	3,01E+01	3,81E+01
4hpi	1,17E+02	1,26E+02	9,82E+01
8hpi	4,04E+02	6,41E+02	3,18E+02
12hpi	2,07E+03	1,20E+03	8,94E+02
24hpi	2,37E+04	1,82E+04	6,64E+03
48hpi	1,15E+05	8,80E+04	6,57E+04
72hpi	1,06E+05	9,82E+04	8,16E+04

Titration on macrophages, HAD50/ml, numbers represent power of ten

	MGF MSV	MGF MSV + nV	MGF nV
-2hpi	0	0	0
0hpi	3,00	2,75	2,75
4hpi	3,50	3,25	3,00
8hpi	4,00	3,50	3,50
12hpi	4,75	4,50	4,25
24hpi	5,75	6,00	5,50
48hpi	7,00	6,50	6,75
72hpi	6,75	7,00	6,75
