# Supplementary material for: Assessment of African swine fever vaccine candidate ASFV-G-∆MGF in a reversion to virulence study
Source: NPJ Vaccines. 2023 May 29;8:78. doi: 10.1038/s41541-023-00669-z (PMC10227017; doi:10.1038/s41541-023-00669-z)
Supplement: Supplementary file 2 — REPORTING SUMMARY [file 41541_2023_669_MOESM2_ESM.pdf]

nature portfolio

Corresponding author(s): Sandra Blome

Last updated by author(s): Jan 28, 2023

Reporting Summary

Nature Portfolio wishes to improve the reproducibility of the work that we publish. This form provides structure for consistency and transparency in reporting. For further information on Nature Portfolio policies, see our Editorial Policies and the Editorial Policy Checklist.

Statistics

For all statistical analyses, confirm that the following items are present in the figure legend, table legend, main text, or Methods section.

n/a	Confirmed	
☒	☐	The exact sample size (*n*) for each experimental group/condition, given as a discrete number and unit of measurement
☒	☐	A statement on whether measurements were taken from distinct samples or whether the same sample was measured repeatedly
☒	☐	The statistical test(s) used AND whether they are one- or two-sided<br>*Only common tests should be described solely by name; describe more complex techniques in the Methods section.*
☒	☐	A description of all covariates tested
☒	☐	A description of any assumptions or corrections, such as tests of normality and adjustment for multiple comparisons
☒	☐	A full description of the statistical parameters including central tendency (e.g. means) or other basic estimates (e.g. regression coefficient) AND variation (e.g. standard deviation) or associated estimates of uncertainty (e.g. confidence intervals)
☒	☐	For null hypothesis testing, the test statistic (e.g. *F*, *t*, *r*) with confidence intervals, effect sizes, degrees of freedom and *P* value noted<br>*Give P values as exact values whenever suitable.*
☒	☐	For Bayesian analysis, information on the choice of priors and Markov chain Monte Carlo settings
☒	☐	For hierarchical and complex designs, identification of the appropriate level for tests and full reporting of outcomes
☒	☐	Estimates of effect sizes (e.g. Cohen's *d*, Pearson's *r*), indicating how they were calculated

Our web collection on statistics for biologists contains articles on many of the points above.

Software and code

Policy information about availability of computer code

Data collection	PCR: Bio-Rad CFX Maestro 1.1 Version 4.1.2433.1219
Data analysis	Real-time PCR and variant detection: Bio-Rad CFX Maestro 1.1 Version 4.1.2433.1219<br>Sequence analysis: Newbler 3.0 (Roche), SPAdes 3.13, and Geneious Prime (2019.2.3, Biomatters)<br>Primers for tailored PCRs were designed using Geneious Prime (Biomatters).<br>Figures: Biorender, GraphPad Prism 8.4.2 (679) for Windows<br>ELISA raw data evalution: Microsoft Excel

For manuscripts utilizing custom algorithms or software that are central to the research but not yet described in published literature, software must be made available to editors and reviewers. We strongly encourage code deposition in a community repository (e.g. GitHub). See the Nature Portfolio guidelines for submitting code & software for further information.

Data

Policy information about availability of data

All manuscripts must include a data availability statement. This statement should provide the following information, where applicable:

- Accession codes, unique identifiers, or web links for publicly available datasets
- A description of any restrictions on data availability
- For clinical datasets or third party data, please ensure that the statement adheres to our policy

All relevant data to support the findings described in the text are included in the main text or in the supplementary materials. Additional data is available from the corresponding author upon reasonable request.

Human research participants

Policy information about studies involving human research participants and Sex and Gender in Research.

Reporting on sex and gender	*Use the terms sex (biological attribute) and gender (shaped by social and cultural circumstances) carefully in order to avoid confusing both terms. Indicate if findings apply to only one sex or gender; describe whether sex and gender were considered in study design whether sex and/or gender was determined based on self-reporting or assigned and methods used. Provide in the source data disaggregated sex and gender data where this information has been collected, and consent has been obtained for sharing of individual-level data; provide overall numbers in this Reporting Summary. Please state if this information has not been collected. Report sex- and gender-based analyses where performed, justify reasons for lack of sex- and gender-based analysis.*
Population characteristics	*Describe the covariate-relevant population characteristics of the human research participants (e.g. age, genotypic information, past and current diagnosis and treatment categories). If you filled out the behavioural & social sciences study design questions and have nothing to add here, write "See above."*
Recruitment	*Describe how participants were recruited. Outline any potential self-selection bias or other biases that may be present and how these are likely to impact results.*
Ethics oversight	*Identify the organization(s) that approved the study protocol.*

Note that full information on the approval of the study protocol must also be provided in the manuscript.

Field-specific reporting

Please select the one below that is the best fit for your research. If you are not sure, read the appropriate sections before making your selection.

☒ Life sciences ☐ Behavioural & social sciences ☐ Ecological, evolutionary & environmental sciences

For a reference copy of the document with all sections, see nature.com/documents/nr-reporting-summary-flat.pdf

Life sciences study design

All studies must disclose on these points even when the disclosure is negative.

Sample size	The design was carried out in compliance with the EMA guideline for testing the reversion risk of veterinary live vaccines in the target species. According to these guidelines, the animal groups should be chosen as small as possible while ensuring statistical power.<br>Assumptions made prior to the trial:<br>Within the first 14 days after immunization with ASFV-G-ΔMGF, reisolation of virus from blood is possible in 3 of 10 animals (data from previous trials with the same vaccine candidate). The expected probability of virus reisolation is therefore set at 0.300. Based on this (reisolation probability 0.300), if the tables in Annex 2 of EMA Guideline VICH GL 41 are used, a reisolation probability of 0.972 can be expected for 10 animals in the first passage. For a group size of 5 animals, the probability of reisolation from at least one animal decreases to 0.832.<br>Therefore, to ensure at least one passenger with sufficient certainty (>95%), the group size should be 10 animals.<br>Sample amounts were based on established in-house protocols considering animal welfare recommendations.
Data exclusions	No data were excluded from analysis.
Replication	Experiments were performed according to best practices and as described in the methods.
Randomization	As only one treatment was performed, randomization of animals was not required.
Blinding	Blinding was not done.

Reporting for specific materials, systems and methods

We require information from authors about some types of materials, experimental systems and methods used in many studies. Here, indicate whether each material, system or method listed is relevant to your study. If you are not sure if a list item applies to your research, read the appropriate section before selecting a response.

Materials & experimental systems

n/a	Involved in the study
☒	☐ Antibodies
☒	☐ Eukaryotic cell lines
☒	☐ Palaeontology and archaeology
☐	☒ Animals and other organisms
☒	☐ Clinical data
☒	☐ Dual use research of concern

Methods

n/a	Involved in the study
☒	☐ ChIP-seq
☒	☐ Flow cytometry
☒	☐ MRI-based neuroimaging

Animals and other research organisms

Policy information about studies involving animals; ARRIVE guidelines recommended for reporting animal research, and Sex and Gender in Research

Laboratory animals	The study comprised domestic pigs, precisely commercial fattening hybrids that were 6-10 weeks old.
Wild animals	*Provide details on animals observed in or captured in the field; report species and age where possible. Describe how animals were caught and transported and what happened to captive animals after the study (if killed, explain why and describe method; if released, say where and when) OR state that the study did not involve wild animals.*
Reporting on sex	African swine fever virus affects male and female animals in the same way. Therefore, reporting of sex was not done. The majority of animals was, however, male.
Field-collected samples	*For laboratory work with field-collected samples, describe all relevant parameters such as housing, maintenance, temperature, photoperiod and end-of-experiment protocol OR state that the study did not involve samples collected from the field.*
Ethics oversight	The animal experiment was approved by the State Office for Agriculture, Food Safety and Fishery in Mecklenburg-Western Pomerania, Germany (LALFF M-V) under reference number 7221.3-1-020/21

Note that full information on the approval of the study protocol must also be provided in the manuscript.
